# Evaluating the vulnerability of *Tetracentron sinense* habitats to climate‐induced latitudinal shifts

**DOI:** 10.1002/ece3.11710

**Published:** 2024-07-14

**Authors:** Yuanjie Gan, Lijun Cheng, Junfeng Tang, Hongyan Han, Xiaohong Gan

**Affiliations:** ^1^ Key Laboratory of Southwest China Wildlife Resources Conservation (Ministry of Education) China West Normal University Nanchong China; ^2^ College of Panda China West Normal University Nanchong China; ^3^ Liziping Giant Panda's Ecology and Conservation Observation and Research Station of Sichuan Province Nanchong China

**Keywords:** climate change, impact factors, MaxEnt, potential suitable distribution area, *Tetracentron sinense*

## Abstract

Exploring the changing process of the geographical distribution pattern of *Tetracentron sinense* Oliv. and its main influencing factors since the last interglacial period can provide a scientific basis for the effective protection and management of the species. The MaxEnt model was used to construct the potential distribution areas of *T. sinense* in different periods such as the last interglacial (LIG), the last glacial maximum (LGM), the mid‐Holocene (MID), and the current and future (2050s and 2070s). On the premise of discussing the influence of dominant environmental factors on its distribution model, the suitable area changes of *T. sinense* under different ecological climate situations were quantitatively analyzed. (1) The AUC and TSS values predicted by the optimized model were 0.959 and 0.835, respectively, indicating a good predictive effect by the MaxEnt model; the potential suitable areas for *T. sinense* in the current period are mainly located in Southwest China, which are wider compared to the actual habitats. (2) Jackknife testing showed that the lowest temperature in the coldest month (Bio6), elevation (Elev), seasonal variation coefficient of temperature (Bio4), and surface calcium carbonate content (T‐CACO_3_) are the dominant environmental factors affecting the distribution of *T. sinense*. (3) From the last interglacial period to the current period, the total suitable area of *T. sinense* showed a decreasing trend; the distribution points of *T. sinense* populations in mid‐Holocene period may be the origin of the postglacial population, and Southwest China may be its glacial biological refuge. (4) Compared with the current period, the total suitable area ranges of *T. sinense* in China in the 2050s and 2070s decreased, and the centroid location of its total fitness area all migrated to the northwest, with the largest migration distance in 2070s under the SSPs 7.0 climate scenario. Temperature was the principal factor influencing the geographical distribution of *T. sinense*. With global warming, the range of *T. sinense* suitable areas will show a shrinking trend, with a shift toward higher‐latitude regions. Ex situ conservation measures could be taken to preserve its germplasm resources.

## Graphical abstract

Temperature was the most important factor affecting the distribution of *Tetracentron sinense*. With global warming, the range of *T. sinense* suitable areas will show a shrinking trend. Ex situ conservation measures could be taken to preserve its germplasm resources.

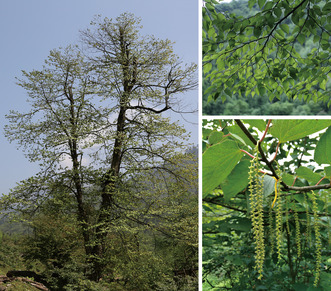

## 1 INTRODUCTION

The geographical distribution of plant populations is influenced by both the biological characteristics of the plant species and their environment (Ge et al., 2012; Thuiller et al., 2005). Primarily, the climate within large‐scale regions serves as the principal determinant affecting species distribution. Changes in climate consequently alter species' responses and selection to climate and habitat (Ma et al., 2013). In recent times, exacerbated by climate change and human interference, species' habitats have suffered severe degradation. This degradation is particularly notable in the significant reduction of suitable growth areas for endangered plants, leading to diminished resources for wild species (Lenoir et al., 2008; Liu et al., 2015). Consequently, investigating the impacts of changing climates on species distribution patterns is crucial for understanding historical and future changes in species range and can furnish a scientific foundation for conserving germplasm resources in endangered plants (Li et al., 2021).

Species distribution models (SDMs) rely on various environmental variables such as climate and soil, closely related to the real growth and distribution of species. These models can predict potentially suitable distribution areas of species using specific algorithms, thereby elucidating the predominant environmental factors influencing their distribution and exploring the ecological requirements of species (Araújo & Peterson, 2012). Among the myriad of models, SDMs encompass 19 different methodologies, including the rule‐set genetic algorithm model (GARP), maximum entropy model (MaxEnt), and ecological factor analysis models (ENFA) (Phillips & Dudik, 2008). The MaxEnt model stands out due to its relative maturity, ease of operation, and high prediction accuracy (Hao et al., 2020). It can infer and predict from incomplete known information, making it widely applicable in studying the introduction and cultivation of relict plants, horticultural tree species, and invasive plants (Elith et al., 2006).

*Tetracentron sinense* Oliv., a Tertiary relict plant, represents the sole surviving species in the *Tetracentron* genus of the Trochodendraceae family (Fan et al., 2021). This species holds significant importance in discussing the systematic evolution of angiosperm plants. Unfortunately, due to its ornamental, furniture, and medicinal value, *T. sinense* has been subjected to extensive exploitation by humans, resulting in poor regeneration of its natural populations (Lu et al., 2020; Pang, 2018; Wang et al., 2023). Therefore, it has been listed as a National Second‐level Key Protected Wild Plant in China (https://www.gov.cn/zhengce/zhengceku/2021‐09/09/content_5636409.htm). The preservation of germplasm resources has garnered considerable attention from researchers (Duan et al., 2020; Zhang et al., 2019).

Fossil records indicate that *Tetracentron* Oliv. was once widely distributed across Europe, North America, and East Asia during the Pleistocene era (Rix & Crane, 2007). A phylogeographical analysis based on the chloroplast genome suggested that the current geographical distribution pattern of *T. sinense* may have been shaped by Quaternary climate oscillations, with Southwest China potentially serving as a biological refuge during glacial periods (Sun et al., 2014). Han et al. (2017) discovered that the phenotypic variation of *T. sinense* is closely correlated with the corresponding environmental factors such as the mean annual sunshine duration, mean temperature in July, and annual mean precipitation. Li (2021) held that low altitude was more conducive to the reproduction, regeneration, and development of *T. sinense* natural populations. In addition, litter depth as well as the soil zinc and calcium content importantly impacted the small‐scale distribution of this species (Zhang et al., 2024). However, the specific influence of these environmental factors on the geographic distribution of *T. sinense* remains ambiguous. How will the distribution pattern of *T. sinense* evolve in the context of past and future climate changes? What are the primary environmental factors constraining its geographical distribution? And how do these factors influence its distribution? These questions remain unanswered, impeding the effective protection and management of *T. sinense* germplasm resources.

Utilizing the MaxEnt model and ArcGIS spatial analysis technology, this study examines potentially suitable areas for *T. sinense* across historical periods (the last interglacial period, the last glacial maximum, and the middle Holocene) as well as current and future periods (2050s and 2070s). The objectives of this study are to (1) analyze the dynamic changes in potentially suitable areas, (2) investigate the main environmental factors driving changes in the distribution pattern of *T. sinense*, and (3) suggest a scientific basis for the effective protection and management of *T. sinense*.

## 2 MATERIALS AND METHODS

### 2.1 Data collection and preprocessing of distribution point data

**FIGURE 1:**
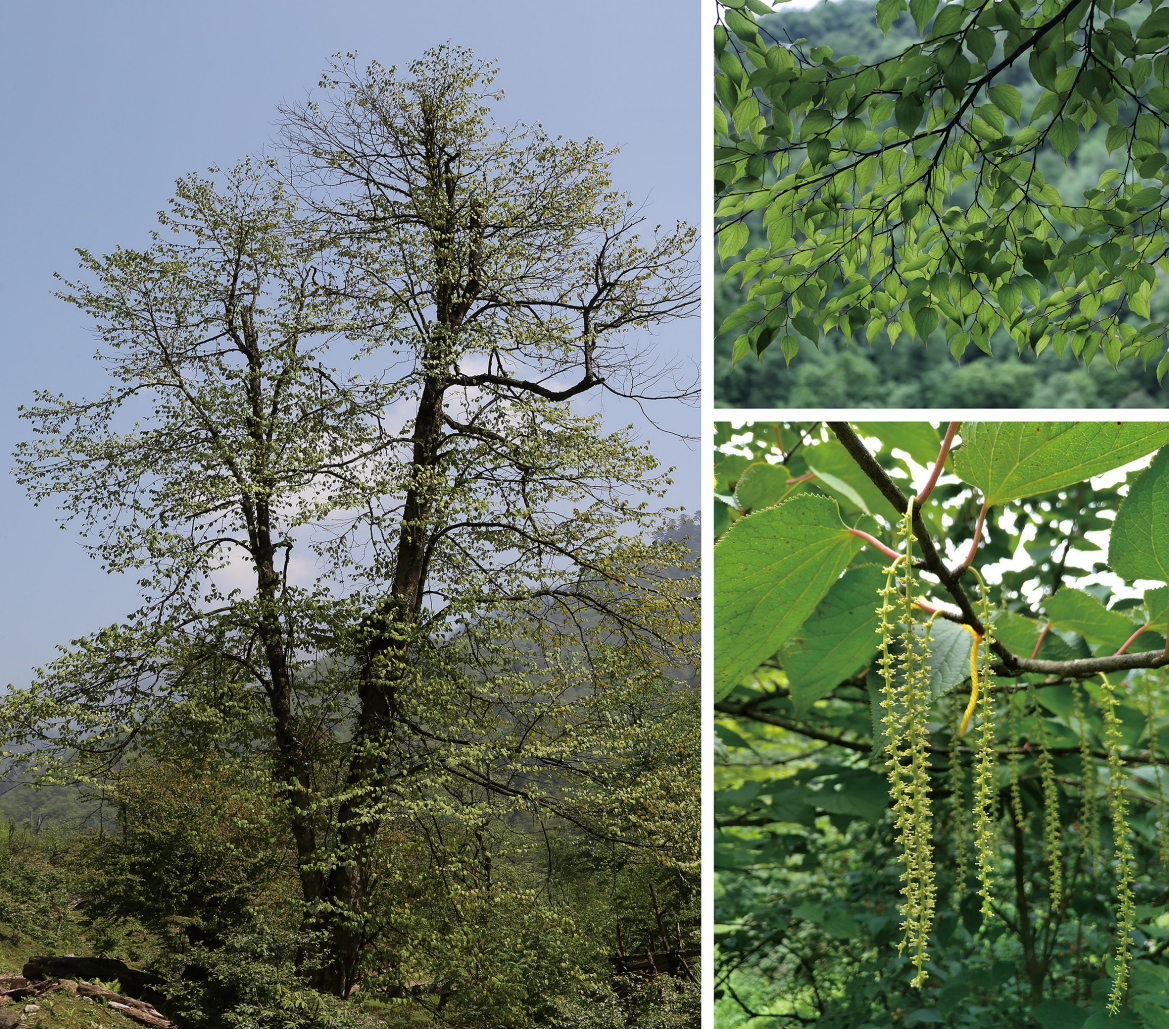
Images of individuals and inflorescences of *Tetracentron sinense* in their natural habitats.

A total of 200 distribution records of *T. sinense* were sourced from various data platforms, including Flora Reipublicae Popularis Sinicae (http://iplant.cn/), Plant Photo Bank of China, and the Global Biodiversity Information Facility (GBIF, http://www.gbif.org). Additionally, our research group conducted field investigations, yielding 121 distribution data points. Consequently, a comprehensive dataset comprising 321 distribution records of *T. sinense* was compiled (Figure 1).

**FIGURE 2:**
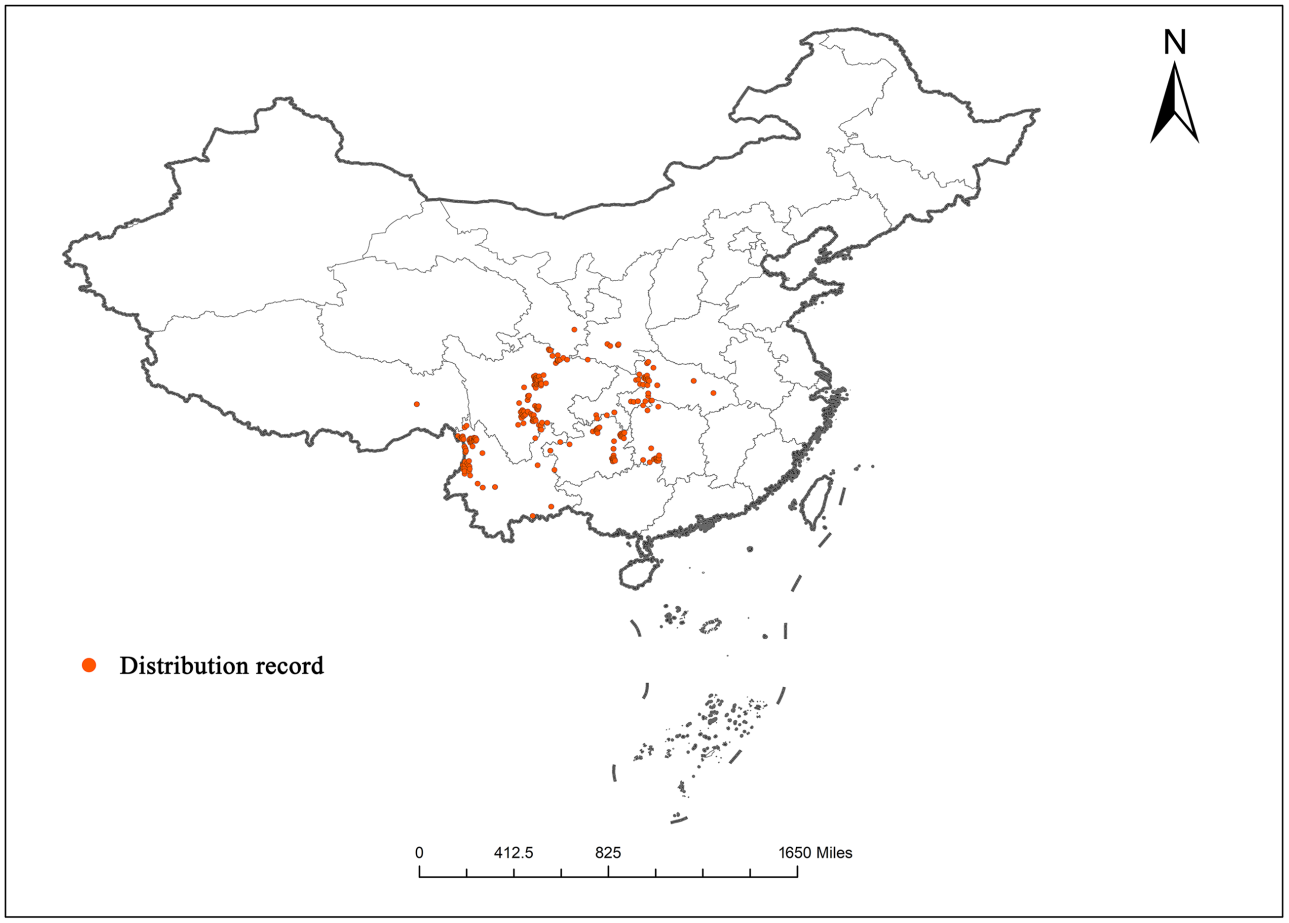
Distribution points of *Tetracentron sinense* after data cleaning.

The coordinate points of *T. sinense*, denoted by latitude and longitude, were converted into decimal numbers compatible with ARCGIS using standard formulas. The resulting table contained three columns: space, longitude, and latitude, and was stored in CSV format. Subsequently, the distribution data in CSV format were imported into ARCGIS 10.2. By utilizing the “Data/Display XY data” and “Data/Export data” functions, a vector file representing the distribution points of *T. sinense* was generated. The projection utilized the WGS1984 geographic coordinate system, with each grid layer retaining only one distribution point. This process ensured an accuracy of 2.5 m, facilitated by ENMTTools software. Following the removal of duplicate entries and the mitigation of sampling deviations, a final set of 233 distribution points was selected (Figure 2).

### 2.2 Environmental variables

We gathered environmental variables associated with bioclimatic, soil, and topographic factors as potential predictors of species distribution (Table 1). Nineteen climate variables spanning the last glacial, last glacial maximum, mid‐Holocene, current periods, and future scenarios were sourced from the World Climate Database (http://worldclim.org) (Gao & Qin, 2018). Future scenarios included low‐concentration emissions (SSPs1‐2.6) and high‐concentration emissions (SSPs5‐8.5) of greenhouse gases. Sixteen soil variables pertaining to the soil surface were acquired from the Chinese Soil Dataset within the Harmonized World Soil Database (HWSD, http://www.fao.org/faostat/en/#data), while elevation data were obtained from the same source. The spatial resolution was set at 2.5 m. Map data were represented in SHP format based on a 1:1‐million‐scale Chinese map obtained from the National Center for Basic Geographic Information.

**TABLE 1:** Environmental variables used in the study.

Type	Variables	Description	Contribution/%
Bioclimatic variables	Bio1	Annual mean temperature	0.1
	Bio2	Mean diurnal range	12.5
	Bio3	Isothermality	5.8
	Bio4	Temperature seasonality	1.5
	Bio5	Max temperature	3.1
	Bio6	Min temperature of coldest month	14.6
	Bio7	Temperature annual range	10.4
	Bio8	Mean temperature of wettest	0
	Bio9	Mean temperature of driest quarter	0
	Bio10	Mean temperature of warmest quarter	1.7
	Bio11	Mean temperature of coldest quarter	14.9
	Bio12	Annual precipitation	3.3
	Bio13	Precipitation of wettest month	0.6
	Bio14	Precipitation of driest month	0
	Bio15	Precipitation seasonality	1.1
	Bio16	Precipitation of wettest quarter	1.4
	Bio17	Precipitation of driest quarter	0.8
	Bio18	Precipitation of warmest quarter	1.3
	Bio19	Precipitation of coldest quarter	1.7
Soil variable	T_GRAVEL	Topsoil gravel content	3.4
	T_SAND	Topsoil sand fraction	0
	T_SILT	Topsoil silt fraction	4.5
	T_CLAY	Topsoil clay fraction	0.1
	T_USDA_TEX_CLASS	Topsoil USDA texture classification	0.8
	T_REF_BULK_DENSITY	Topsoil reference bulk density	0.3
	T_OC	Topsoil organic carbon	0.1
	T_PH_H2O	Topsoil pH (H2O)	0.3
	T‐ESP	Topsoil sodicity (ESP)	0
	T_CEC_CLAY	Topsoil CEC (clay)	0.8
	T_BS	Topsoil base saturation	2.2
	T_TEB	Topsoil TEB	0.2
	T_CACO3	Topsoil calcium carbonate	4.7
	T_CASO4	Topsoil gypsum	0
	T_ECE	Topsoil salinity (Elco)	0
	T_CEC_SOIL	Topsoil CEC (soil)	0.1
Topography	Elev	Elevation	7.5

To mitigate multicollinearity and potential model overfitting (Graham, 2003), we conducted Spearman's correlation analysis within ArcGIS (Yang et al., 2013) to examine relationships among environmental factors. Variables demonstrating a correlation coefficient |*r*| ≥ .8 were considered highly correlated, and the less influential factor was excluded from subsequent analysis. Consequently, a total of 16 environmental factors were retained for calculation and analysis within the MaxEnt model.

Climate and elevation variable data were clipped according to the vectograph of a 1:1‐million‐scale Chinese administrative map and then converted to ASC format using ArcGIS software. Soil variable data were integrated by importing the China soil file and HWSD DATA file into ArcGIS software. Subsequently, the grid layer comprising the 16 soil variables within the MU_GLOBAL layer was extracted and converted into ASC format. Finally, all environmental variable layers underwent batch processing using ArcGIS software, resulting in environment layers with non‐overlapping extents.

### 2.3 Model building, optimization, and evaluation

In accordance with the latest model optimization methodology proposed by Cobos et al. (2019), adjustments were made to the parameters of the MaxEnt model to assess the degree of alignment between the distribution points of *T. sinense* and the model, as determined by the corrected Akaike information criterion (AICc) (Yu et al., 2019). Optimal model parameters, indicated by the lowest AICc value, were selected for utilization within the MaxEnt software (Philips et al., 2017).

The distribution data of *T. sinense* and its corresponding environmental variables spanning the study period were inputted into the MaxEnt model. In this investigation, 25% of the 232 distribution sites of *T. sinense* were earmarked for model evaluation, while the remaining 75% constituted the training set for constructing a response curve. Ten replicates were generated for each training partition, and the resultant outcomes were averaged. Model results were generated in both logistic and ASC format files, with a multifeature combination based on optimization outcomes (Moreno et al., 2011).

To calibrate the model and assess its robustness, the prediction accuracy of the MaxEnt model was evaluated using the area under the curve (AUC) and the true skill statistic (TSS) (Xu et al., 2019). The AUC values ranged between 0 and 1, with higher values indicating higher prediction accuracy. The AUC values were classified into five categories as follows: unqualified (0.5–0.6), poor (0.6–0.7), average (0.7–0.8), good (0.8–0.9), and excellent (0.9–1.0) (Fithian et al., 2015). The TSS values ranged between −1 and 1, with values closer to 1 indicating better prediction performances. A TSS value between 0.6 and 1 suggests a good prediction capability (Allouche & Kadmon, 2006).

### 2.4 Classification of fitness levels and area statistics

The average output data from each period, following 10 simulations in the MaxEnt model, were imported into ArcGIS software. These data were then converted into raster layers and subsequently reclassified based on the distributed probability (*p*) values. Employing natural breakpoint classification, the distribution area of *T. sinense* was categorized into four distinct levels: non‐suitable area (*p* < .2), low suitable zone (.2 ≤ *p* < .4), intermediate suitable zone (.4 ≤ *p* < .6), and high suitable zone (*p* ≥ .6). The reclassified layers were processed using an ArcGIS grid table to determine the area encompassed by each level (Zhuang et al., 2018).

### 2.5 Screening and threshold analysis of dominant climate factors

We conducted an analysis to identify the primary environmental factors influencing the distribution of *T. sinense*. This analysis relied on assessing the permutation importance (PI) and permutation contribution (PC) of environmental variables within the prediction results (Mbari, 2020).

### 2.6 Spatial pattern change

The distribution probability of *T. sinense* underwent reclassification, and a grid calculator was employed to delineate the distribution change layer of *T. sinense* between historical or future periods and current climate scenarios. Based on the grid values, four types of distribution area changes in *T. sinense* were redefined: retention (3), additions (2), losses (1), and non‐suitable (0). The suitable area for each type was determined by calculating the proportion of each value (Wang et al., 2024).

## 3 RESULTS

### 3.1 Species distribution model and its accuracy

Utilizing 233 geographic distribution data points and 36 environmental variables, we employed the MaxEnt model to simulate and predict potentially suitable areas for *T. sinense*. Optimization using the Emavel data package revealed that when the feature combination (FC) was set to LP and the regularization multiplier (RM) to 0.9, an AICc value of 0 indicated optimal predictive performance. Hence, FC = LP and RM = 0.9 were chosen as the final parameter settings (Table 2).

**TABLE 2:** Model parameter.

Model evaluation	Feature combination	Regularization multiplier	Value of delta Akaike information criterion corrected	5% Training omission
Default	LQHPT	1	259.485332	0.103
Optimized	LP	0.9	0	0.034

**FIGURE 3:**
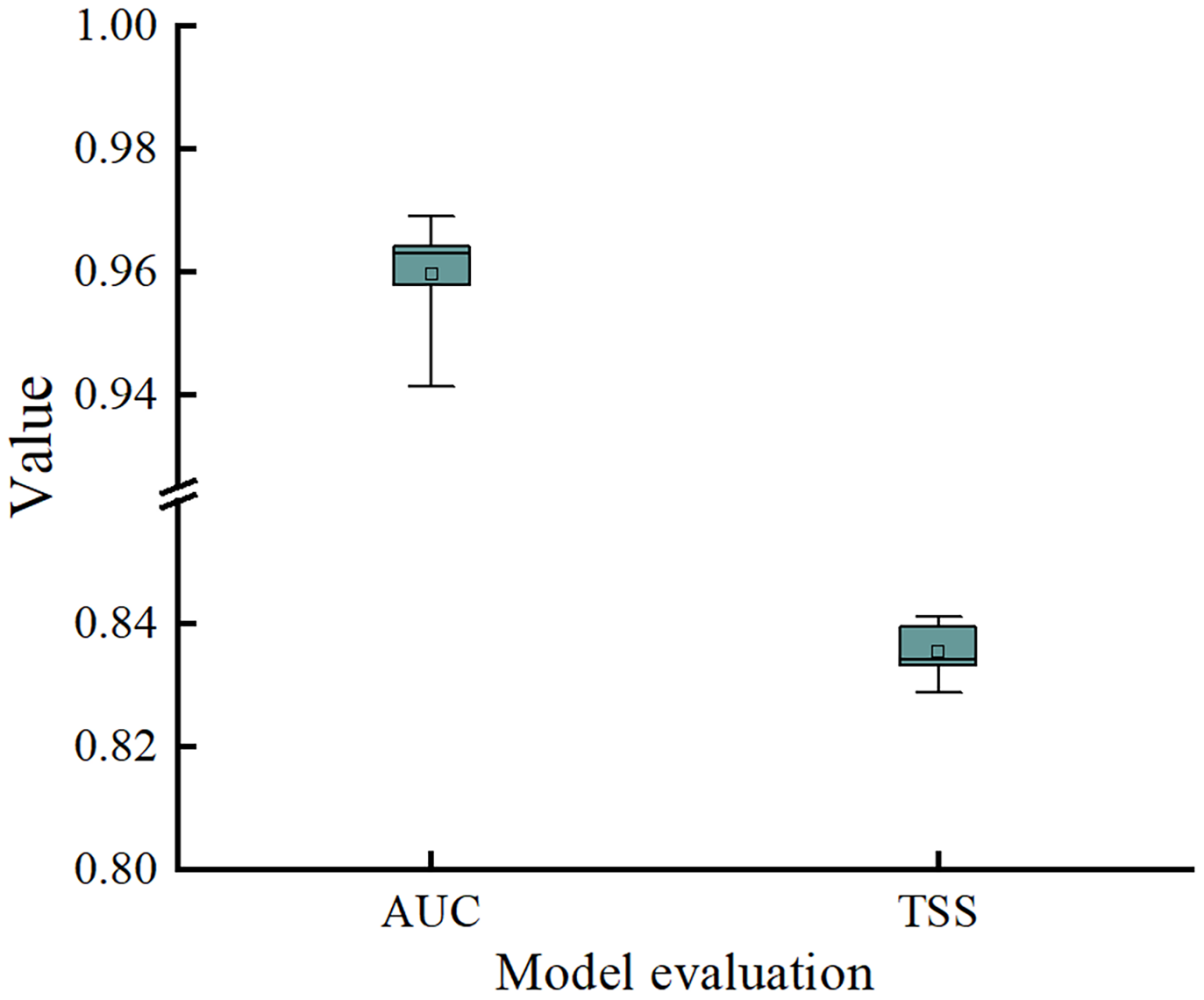
Model evaluation results of AUC value and TSS value.

In this study, MaxEnt was executed with optimal model parameter settings and subjected to 10‐fold cross‐validation, resulting in an average AUC and TSS of 0.959 and 0.835, respectively (Figure 3). These values indicated that the MaxEnt model could be applied to simulate and predict the distribution of suitable areas for *T. sinense*.

### 3.2 Importance of environmental variables affecting *T. sinense* distribution

**FIGURE 4:**
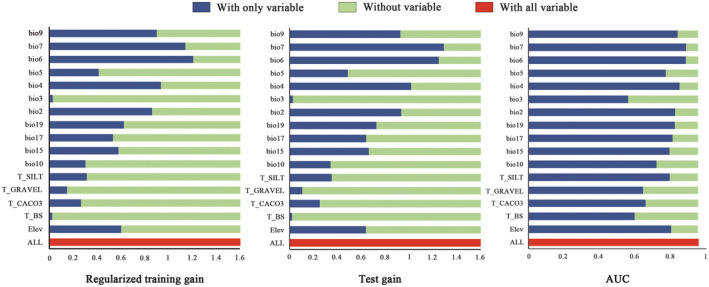
The jackknife test result for the environmental factors.

The influence of 16 environmental factors on distribution was evaluated through a jackknife test (Figure 4). Notably, the contribution rates (PC) of Bio6 (44.60%), T_CACO3 (10.29%), and Bio9 (8.90%) ranked highest, with a cumulative PC of 63.79%. Similarly, the permutation importance (PI) of Bio6 (34.18%), Bio4 (25.70%), and elevation (21.18%) were among the top three, with a cumulative PI of 81.06%. This analysis revealed that the primary environmental factors shaping the current geographical distribution of *T. sinense* include bioclimatic variables (Bio4 and Bio6), soil variables (T_CACO3), and topography (Elevation) (Table 3).

**TABLE 3:** Main parameters of environmental factors.

Variable	PC	PI	RTGw	RTGo	TGw	TGo	AUCw	AUCo
bio6	44.6012	34.1752	2.1863	1.2083	2.2081	1.2511	0.9592	0.8887
T_CACO3	10.2886	2.4837	2.1824	0.268	2.2161	0.2534	0.9592	0.6611
bio9	8.8969	0.5763	2.1975	0.9028	2.218	0.9267	0.9595	0.8435
Elev	7.1365	21.1835	2.1492	0.6041	2.167	0.6384	0.9573	0.8059
bio2	6.8383	0.036	2.1981	0.8624	2.2208	0.9352	0.9596	0.8285
bio10	5.0883	0.0595	2.1982	0.3042	2.2196	0.3437	0.9595	0.723
T_GRAVEL	3.4221	0.1516	2.1925	0.1493	2.2205	0.1051	0.9597	0.6468
bio7	3.0458	0.1643	2.1981	1.1432	2.218	1.2907	0.9595	0.8902
bio4	2.9022	25.6958	2.1867	0.9363	2.2065	1.0171	0.9592	0.8546
T_SILT	2.288	1.5817	2.1681	0.3146	2.1914	0.352	0.9581	0.7978
bio5	1.4892	0.0014	2.1982	0.4146	2.2185	0.4884	0.9595	0.7767
bio15	1.2819	1.5014	2.1853	0.5794	2.2109	0.6629	0.9595	0.7961
bio3	1.1263	6.6682	2.1787	0.031	2.2096	0.0282	0.9593	0.5629
T_BS	0.7965	0.1273	2.1921	0.025	2.216	0.0211	0.9595	0.599
bio19	0.5863	3.4403	2.1792	0.6257	2.1864	0.7266	0.9581	0.8264
bio17	0.2119	2.1538	2.1901	0.535	2.2044	0.6404	0.9589	0.8139

**FIGURE 5:**
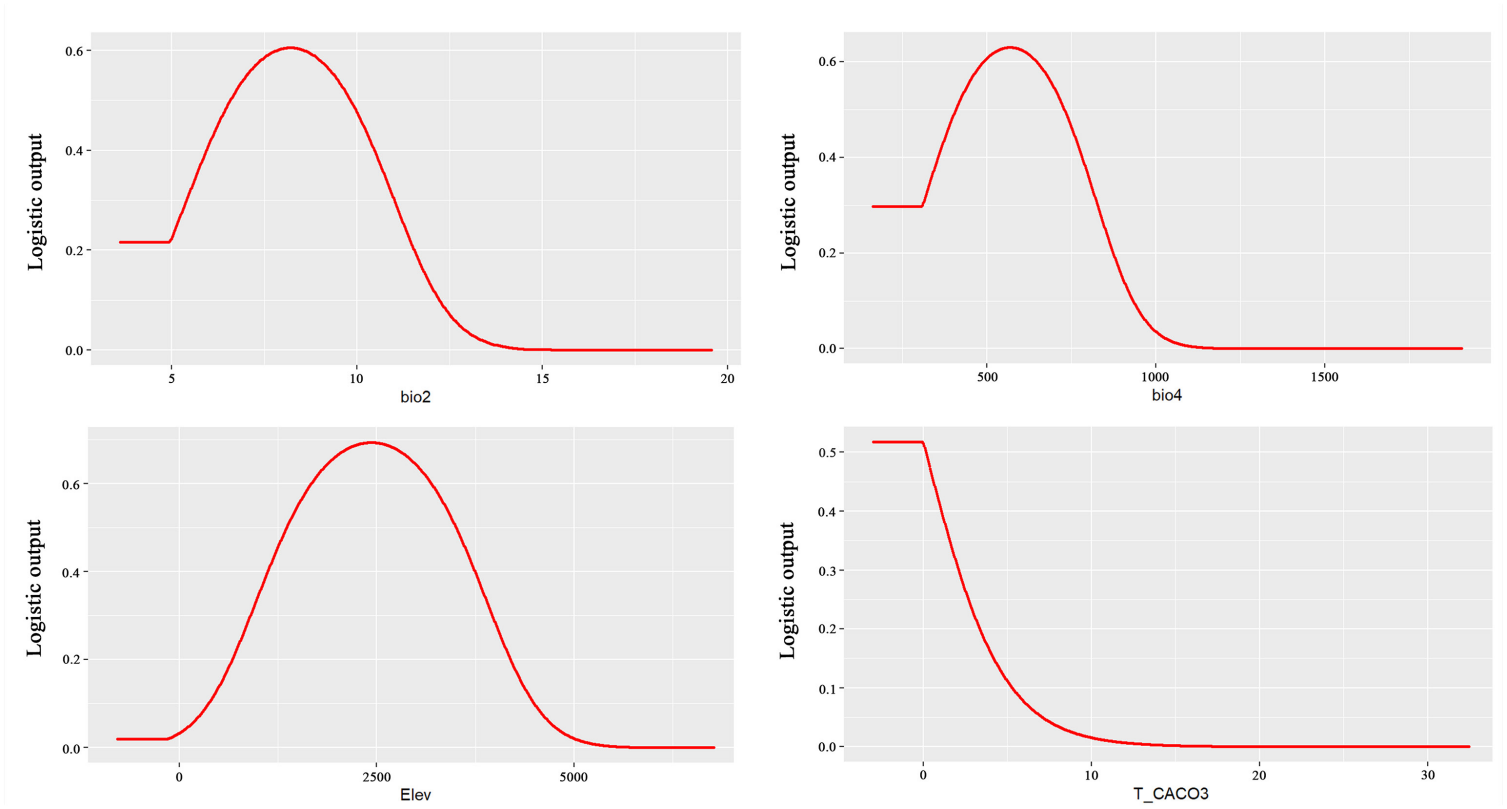
Response curves of dominant environmental factors.

Environmental factor response curves further elucidated the relationship between the probability of *T. sinense* occurrence and environmental variables (Figure 5). Generally, when the probability exceeded 0.5, corresponding environmental factor values favored *T. sinense* growth (Li et al., 2022). Based on these curves, suitable environmental factor ranges for *T. sinense* growth were determined as follows: 407.4 to 731.3 (Bio4), −8 to 0.61°C (Bio6), 0% to 0.17% (T_CACO3), and 1355.8 to 3508 m (elevation).

### 3.3 Suitable distribution areas for *T. sinense* under current climatic conditions

**FIGURE 6:**
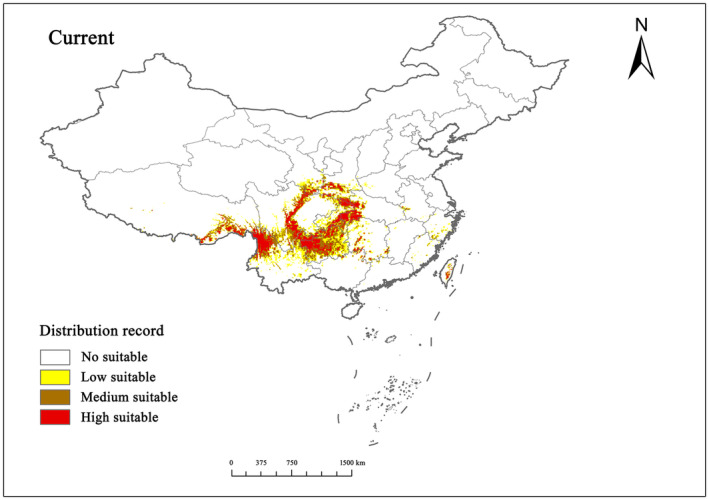
Suitable distribution of *Tetracentron sinense* in China under current climatic conditions.

Currently, *T. sinense* finds suitable habitat across approximately 7.24% of China's total land area. Central Sichuan, Northwestern Yunnan, Western Guizhou, and Southwestern Hubei emerge as primary distribution hubs, with suitable areas radiating outward in distinct patterns (Figure 6). Notably, highly suitable areas connect with moderately suitable regions, while less suitable areas extend from them. Compared to the current distribution, new suitable areas, such as Zhejiang, Anhui, Fujian, Henan, Guangdong, and Taiwan, have emerged along coastal regions. Expansion of *T. sinense* distribution is observed in provinces such as Chongqing, Guizhou, and Hunan, while the species' range remains largely unchanged in other regions.

### 3.4 Prediction of suitable areas of *T. sinense* in historical and future climates

This study analyzed six periods to predict potential distribution areas for *T. sinense*. Optimal distribution areas varied across periods, predominantly favoring Southwest China.

**FIGURE 7:**
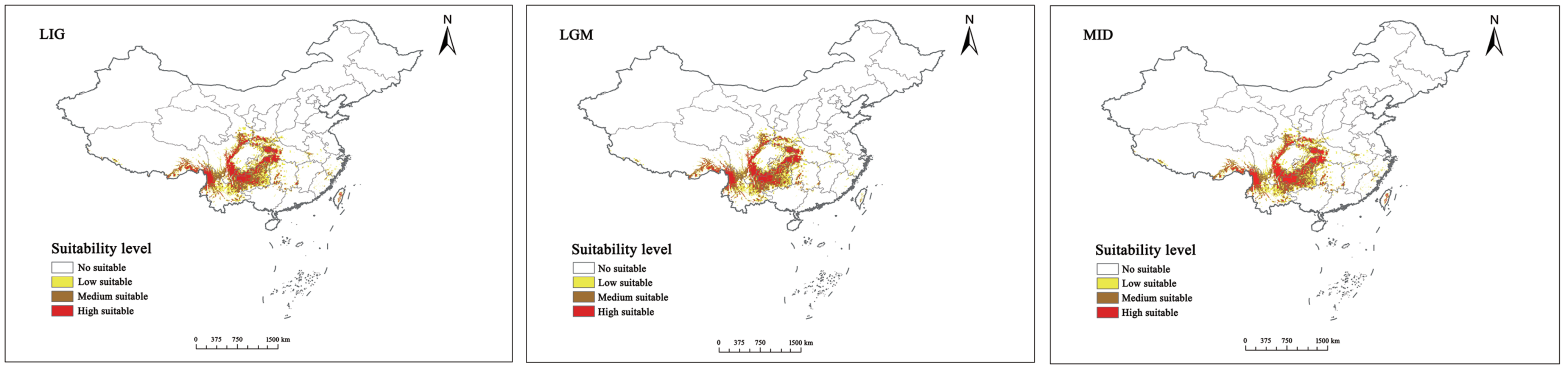
Prediction of potentially suitable areas of *Tetracentron sinense* in historical period. *Note*: LGM, last glacial maximum; LIG, last interglacial; MID, Mid‐Holocene; 50s‐126 ~ 50s‐585 were SSPs1‐2.6, SSPs2‐4.5, SSPs3‐7.0, and SSPs5‐8.5 of 2050s, respectively; and 70s‐126 ~ 70s‐585 were SSPs1‐2.6, SSPs2‐4.5, SSPs3‐7.0, and SSPs5‐8.5 of 2070s, respectively. The same as below.

The total suitable area for *T. sinense* has exhibited fluctuations from the last interglacial period to the present (Figure 7). Initially, it stood at 758,258 km^2^, decreasing to 731,469.3 km^2^, then rising to 750,132.8 km^2^ before sharply declining to 675,550.8 km^2^. Similar trends were observed in high, medium, and low suitability areas.

**FIGURE 8:**
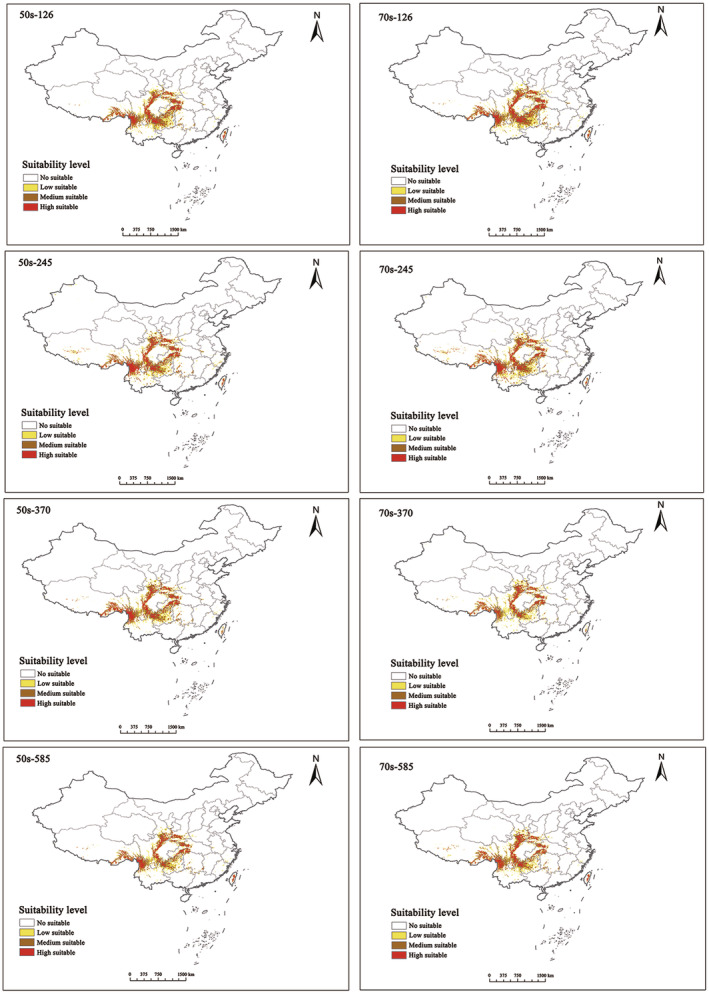
Prediction of potentially suitable areas of *Tetracentron sinense* in future period.

Under various climate scenarios for the 2050s and 2070s, significant changes occurred in suitable areas due to climate shifts (Figure 8). In the 2050s, the climate scenario yielding the largest suitable area was SSPs2‐4.5, which was 60,955.2 km^2^ less than the current scenario. By 2070, SSPs1‐2.6 exhibited the largest suitable area, 39,032.8 km^2^ less than the current scenario. Across all grades, the future suitable habitat area for *T. sinense* is expected to decrease compared to the current area.

### 3.5 Changes in spatial pattern of potential suitable areas of *T. sinense*

**FIGURE 9:**
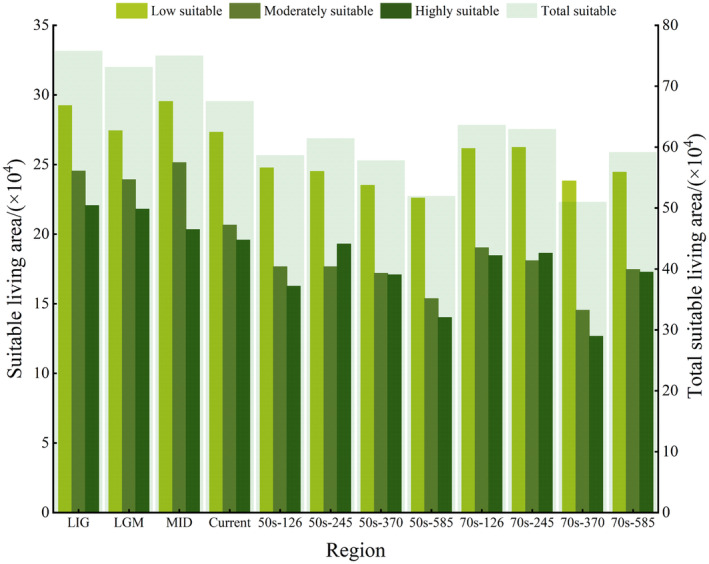
Comparison of suitable distribution areas in different periods.

**FIGURE 10:**
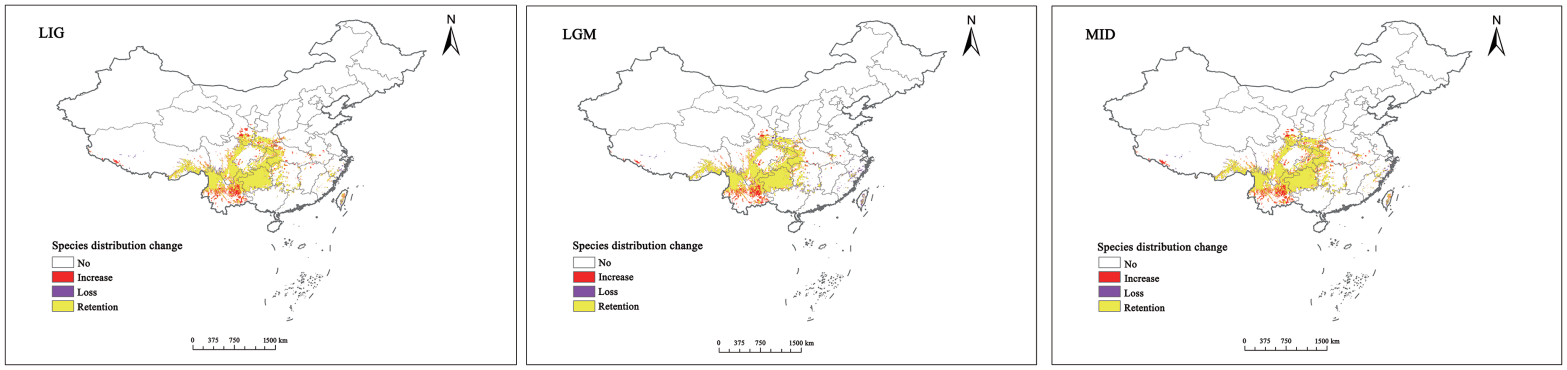
Spatial pattern changes of *Tetracentron sinense* under historical climate change scenarios.

Compared to the current period, the suitable area for *T. sinense* decreased from the last interglacial period to the mid‐Holocene, followed by an increase (Figure 9). During the mid‐Holocene, an additional area of approximately 119,693.5 km^2^ emerged, constituting 17.72% of the total area. These additions were mainly concentrated in Central Yunnan and Southern Gansu. Conversely, during the last glacial period, the cold climate led to a significant reduction in suitable habitats, resulting in a loss of 42,620.2 km^2^, or a 6.3% decrease (Table 4). Losses occurred predominantly in fragmented areas at the junction of Sichuan, Yunnan, Gansu, and Shaanxi provinces. Retention areas during the last glacial maximum were primarily situated in Southwest China (Figure 10).

**TABLE 4:** Spatial changes in suitable areas of *Tetracentron sinense* under different climate change scenarios.

Period	Area/km^2^				Area change rate/%			
	Additio*n*	Loss	Retention	Change	Increasing rate	Attrition rate	Retention rate	Total
LIG	111,055	28,374.16	647,234	82,680.79	16.44	4.20	95.81	108.05
LGM	98,509.7	42,620.18	632,988	55,889.52	14.58	6.31	93.70	101.97
MID	119,693.5	29,653.95	645,954.2	90,039.55	17.72	4.39	95.62	108.95
50s‐126	32,884.35	121,586.2	553,949	−88,701.9	4.87	18.00	82.00	68.87
50s‐245	77,403.53	138,390.2	537,160.6	−60,986.6	11.46	20.49	79.51	70.49
50s‐370	61,542.51	159,033.9	516,485.6	−97,491.4	9.11	23.54	76.45	62.02
50s‐585	47,145.94	203,047.5	472,503.4	−155,902	6.98	30.06	69.94	46.87
70s‐126	48,964.43	88,028.21	587,522.6	−39,063.8	7.25	13.03	86.97	81.19
70s‐245	99,629.48	145,428.4	530,038.2	−45,798.9	14.75	21.53	78.46	71.68
70s‐370	78,531.53	243,828.3	431,704.5	−165,297	11.62	36.09	63.90	39.44
70s‐585	113,049.2	196,851.1	478,699.7	−83,801.9	16.73	29.14	70.86	58.46

**FIGURE 11:**
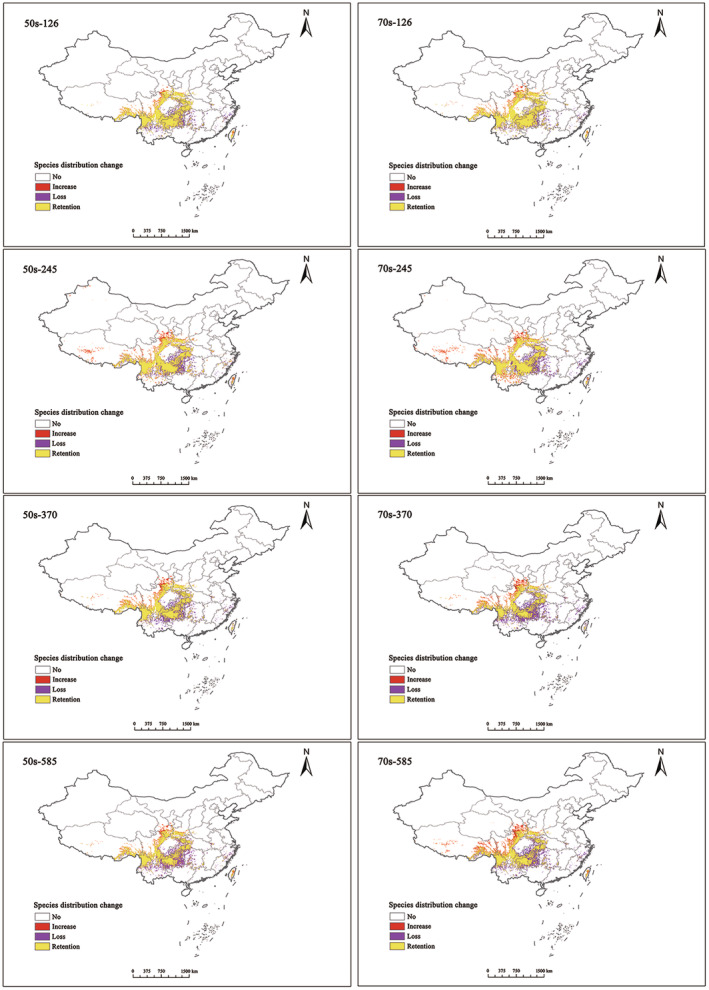
Spatial pattern changes of *Tetracentron sinense* under future climate change scenarios.

The expansion rate for 2050 was lower than that for 2070 across eight different climate scenarios with similar concentrations (Figure 11). In the 2050s, the additional area for *T. sinense* initially expanded, then contracted with increasing greenhouse gas emissions. Conversely, by the 2070s, the additional area exhibited an upward trend with increased emissions. Except for the SSPs2.6 scenario, the loss rate in the 2070s was significantly higher than that in the 2050s (Table 4). Overall, under future climate scenarios, fragmentation of potential distribution areas suitable for *T. sinense* is expected to increase. Loss areas will concentrate in the southern region of the current suitable area, while added areas will primarily occur in the north. These areas require close monitoring for potential pattern changes.

### 3.6 Core distributional shifts

**FIGURE 12:**
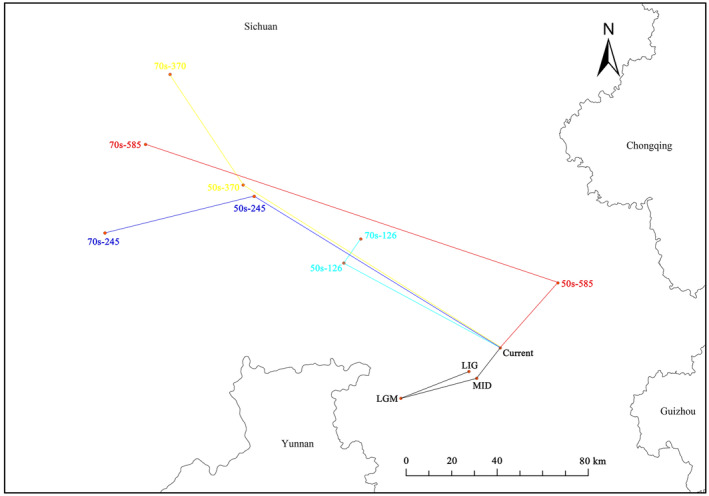
The core distributional shifts of suitable habitat under different climate scenarios for *Tetracentron sinense*.

Throughout history, the centroid of *T. sinense* has exhibited notable fluctuations (Figure 12). From the last interglacial period to the last glacial maximum, the center of mass shifted southwest by 49,638 m. Subsequently, from the last glacial period to the mid‐Holocene, the centroid moved northeast by 36,430 m. Continuing into the present era, the center of mass further migrated northeast by 18,472.6 m.

Projection analysis indicates a northward shift in the centroid of the suitable area for *T. sinense* by 2050 and 2070, under future climate change scenarios (Figure 12). With increasing greenhouse gas emissions, the spatial distribution of potentially suitable areas undergoes more pronounced alterations over greater distances. Notably, the migration distance of *T. sinense* in the 2070s surpasses that of the 2050s under low and medium emission concentrations, except for the center of the 2050s‐SSPs8.5. The most extensive migration distance occurs under the SSPs7.0–2070s climate scenario.

## 4 DISCUSSION

### 4.1 Environmental factors affecting *T. sinense* distribution

Results from the jackknife test and analysis of the main parameter table underscore that the potential distribution of *T. sinense* is influenced by four key environmental factors: Bio4, Bio6, T_CACO3, and elevation. Elevation plays a significant role in species distribution by indirectly affecting temperature and precipitation (Clark & Husband, 2007; Ma, Guo, et al., 2021; Ma, Lu, et al., 2021). While topsoil calcium carbonate does impose some restrictions on plant distribution, its impact appears less pronounced (Zhang et al., 2024). Therefore, temperature emerges as the primary factor shaping the geographical distribution of *T. sinense*, a finding corroborated by previous studies. Li et al. (2021), for instance, employed the concept of space–time substitution to investigate the influence of altitude on the reproductive characteristics of *T. sinense*, proposing that temperature variations impact species fitness and could prompt migration to higher altitudes with rising temperatures. Similarly, Chen et al. (2023) through correlation analysis of chronological and meteorological factors identified air temperature during specific periods as a key influencer during *T. sinense* growth stages. In general, temperature fluctuations can indirectly affect the physiological and metabolic activities of *T. sinense* by influencing their leaf phenotype, respiration, photosynthesis, and water absorption capacity. Consequently, these effects would influence *T. sinense* survival, reproduction, and growth, ultimately impacting importantly its distribution (Han et al., 2017; Li et al., 2020).

The permutation importance (PI) and jackknife tests further highlight the critical role of the minimum temperature of the coldest month (bio6) in shaping the potential geographical distribution of *T. sinense*. This finding aligns with observations in other species such as *Quercus mongolica* (Yin et al., 2013), *Santalum album* (Hu et al., 2014), *Gymnocarpos przewalskii* (Zhao et al., 2020), and *Thuja sutchuenensis* (Ma, Guo, et al., 2021; Ma, Lu, et al., 2021). Chen's research also revealed a significant negative correlation between *T. sinense* growth and the lowest temperatures in November. Consequently, the minimum temperature during the coldest month emerges as a pivotal factor constraining northward expansion of *T. sinense*. Low temperatures not only hinder seed germination and morphological development but also pose challenges to the species' cold resistance, ultimately impeding its normal growth and development in Northern China.

### 4.2 Changes in the potential geographical distribution of *T. sinense*

Our investigation revealed fluctuations in the total suitable area of *T. sinense*, which decreased from 758,258 km^2^ to 731,469.3 km^2^ from the last interglacial period to the last glacial maximum. During this period, suitable habitats contracted, primarily concentrating in the central part of Southwest China. The mountainous terrain in this region acted as a barrier against cold air, mitigating extreme climate fluctuations and providing stable conditions conducive to species survival (Li, 2017; Stewart et al., 2010). Furthermore, the absence of geographical barriers facilitated migration to this area, establishing Southwest China as a critical refuge for the tertiary relict *T. sinense*, consistent with the results from Sun et al. (2014). The refuges can buffer ice‐age climates, help *T. sinense* through harsh environmental conditions, and provide a source for the species to re‐enter other habitats (Chen, Hill, et al., 2011; Chen, Kang, et al., 2011; Liang, 2020). Subsequently, from the last glacial maximum to the mid‐Holocene, the total suitable area expanded to 750,132.8 km^2^, with suitable habitat extending outward from the Sichuan Basin and Yunnan–Guizhou Plateau. This expansion correlates with the warmer and wetter global climate during the mid‐Holocene, aligning with the hydrothermal conditions favorable for *T. sinense* growth. Consequently, the population of *T. sinense* exhibited significant glacial contraction and post‐glacial expansion, consistent with findings for other species such as *Thuja sutchuenensis* (Qin et al., 2017), *Davidia involucrata* (Ye et al., 2021), and *Ulmus elongata* (Zhang et al., 2021).

From the mid‐Holocene to our current time, the total suitable area for *T. sinense* reduced by 74,582.0 km^2^, potentially attributed to the intensified human activities such as increased contemporary energy consumption, amplified carbon dioxide emissions, deforestation, and agricultural land expansion (Ye et al., 2021), resulting in *T. sinense* habitat fragmentation. However, we observed that the currently predicted suitable habitat area exceeds the actual habitat area, a discrepancy potentially attributable to several factors. First, the modeling dataset predominantly comprises data from earlier periods, with scant recent distribution data. Second, the native habitat of *T. sinense* wild populations is mainly concentrated in the southwestern region of China, marked by diverse climate conditions and vegetation types. Hence, the probability of undiscovered distribution points within these intricate ecosystems is notably high.

In the future, global climate warming is anticipated to substantially impact suitable habitats for *T. sinense*, resulting in a significant distribution shift. The extent of this shift varies depending on emission scenarios, with the largest loss area observed in the SSPs8.5 scenario and the smallest in the SSPs2.6 scenario. This disparity is attributed to temperature surpassing the threshold required for optimal *T. sinense* growth in the SSPs8.5 scenario. Consequently, temperature increase emerges as a primary driver of future reductions in suitable distribution areas. Therefore, the reduction of suitable habitat areas for *T. sinense* in the future will increase its risk of extinction, posing challenges to the continued survival and reproduction of its natural populations.

The centroid analysis revealed that the future distribution of *T. sinense* is shifting northwestward, toward higher latitudes, a trend consistent with that for various other species (Chen, Hill, et al., 2011; Chen, Kang, et al., 2011). This migration pattern could lead to the original habitat loss of the species coupled with population size reduction. Moreover, as *T. sinense* enters new habitats, it will encounter intense interspecific competition with new coexisting species. Its limited adaptation and weaker photosynthetic capacities compared to those of coexisting species would likely represent a disadvantage during interspecific competitions, thereby hindering its ability to survive and reproduce in new habitats (Yang, 2023).

### 4.3 Protection and management strategy

Our findings underscore a concerning trend: under future climate conditions, the rate of decline in *T. sinense* populations is projected to surpass the rate of expansion, posing a significant risk of extinction. Urgent action is therefore warranted to address this potential survival crisis. Identification of the primary potential distribution regions of *T. sinense* enables the strategic establishment of nature reserves in these areas, crucial for safeguarding the natural habitats of wild *T. sinense* populations.

Currently, the main potential distribution regions of *T. sinense* have been identified, enabling the designation of nature reserves in these areas to bolster the safeguarding of the natural habitats of wild *T. sinense*. The suitable habitat for *T. sinense* would expectably shift toward higher‐latitude regions in the future. Therefore, moderate northwest movement beyond the existing predicted areas is imperative for nature reserve designation. Given the habitat loss, it is imperative to consider the impacts on associated species when devising ex situ conservation strategies. Moreover, proactive measures tailored to the growth requirements of *T. sinense* should be implemented in newly identified areas to mitigate potential disruptions. Preserving retention areas can serve as secure sanctuaries, allowing trees to adapt to climate change. Thus, it is essential to bolster the protection and management efforts in these critical zones.

### 4.4 Limitations and future perspectives

Although the MaxEnt model is widely used in species distribution prediction and demonstrates good performance in the case of incomplete data and small sample sizes, its accuracy might be potentially compromised when few species distribution points or inaccurate environmental variables are available (Phillips et al., 2006). Multiple studies described that constructing small ensemble models (ESMs) based on generalized linear models (GLM), gradient boosting machines (GBM), and MaxEnt could yield modeling results superior to those of individual species distribution models, effectively overcoming the limitations of single models in the modeling of rare species (Breiner et al., 2015). Furthermore, if the MaxEnt model only considers abiotic factors, the resulting measurements might also be subject to certain degree of error. Therefore, generalized linear models (GLM) and gradient boosting machines (GBM) could be combined in the future, taking into account various factors such as biotic factors as well as intra‐species and inter‐species relationships (Zhou et al., 2023), and even certain anthropogenic factors (Feeley & Silman, 2010) to enhance the accuracy of species distribution prediction and better elucidate the changing geographical distribution patterns of *T. sinense*.

Temperature has been identified as the primary factor determining *T. sinense* distribution, marking the first step toward understanding its response to global warming. Future research should focus on how temperature impacts *T. sinense* population dynamics, growth, reproduction, adaptation, and ecosystem function under different climatic conditions. Supporting long‐term monitoring and ecosystem dynamics research is crucial for understanding the ecological and evolutionary effects of temperature and developing climate change adaptation strategies for *T. sinense*.

## 5 CONCLUSIONS

This study employed the MaxEnt model to delineate the suitable distribution areas of *T. sinense* across different temporal periods. Our analysis identified four key environmental factors—Bio4, Bio6, T_CACO3, and elevation—as primary determinants shaping the potential distribution of *T. sinense*. Specifically, temperature emerged as the principal factor influencing the geographical distribution of *T. sinense*.

The dynamic changes in suitable areas for *T. sinense* during historical periods adhered to the pattern of “glacial contraction and postglacial expansion.” However, in the context of global warming, our findings reveal a concerning trend of decreasing suitability for *T. sinense*, with a shift toward higher‐latitude regions.

These results furnish a robust scientific foundation for guiding the management, conservation, and judicious site selection strategies aimed at preserving *T. sinense* populations in the face of ongoing environmental changes. Furthermore, they provide novel insights into the future changes of rare species facing climate change, thereby offering theoretical guidance for endangered plant conservation and references for biodiversity conservation.

## AUTHOR CONTRIBUTIONS

**Yuanjie Gan:** Conceptualization (equal); data curation (equal); methodology (equal); software (equal); writing – original draft (equal). **Lijun Cheng:** Data curation (equal); software (equal); visualization (equal). **Junfeng Tang:** Methodology (equal); software (equal). **Hongyan Han:** Data curation (equal); software (equal); visualization (equal). **Xiaohong Gan:** Data curation (equal); project administration (equal); supervision (equal); visualization (equal); writing – review and editing (equal).

## FUNDING INFORMATION

This study was supported by the National Natural Science Foundation of China (No. 32070371), the Innovation Team Funds of China West Normal University (No. KCXTD2022‐4), the Sichuan Meigu Dafengding National Nature Reserve (No. mgdfd2022‐13), Sichuan Micang Mountain National Nature Reserve (No. N5108212022000043), and Natural Science Foundation of Sichuan Province (No. 23NSFSC1272).

## CONFLICT OF INTEREST STATEMENT

The authors declare no competing interests.

## Data Availability

Datasets used in this study are available online from the Dryad Digital Repository (https://datadryad.org/stash/share/osQnAcuL0yh0p3g4z78YVAhXCRIoPeEDpRev5Xjc_jE).
